# Systematic analysis of protein identity between Zika virus and other arthropod-borne viruses

**DOI:** 10.2471/BLT.16.182105

**Published:** 2016-07-18

**Authors:** Hsiao-Han Chang, Roland G Huber, Peter J Bond, Yonatan H Grad, David Camerini, Sebastian Maurer-Stroh, Marc Lipsitch

**Affiliations:** a Department of Epidemiology, Center for Communicable Disease Dynamics, Harvard TH Chan School of Public Health, 677 Huntington Ave, Boston, Massachusetts, 02115, United States of America (USA).; b Bioinformatics Institute (BII), Agency for Science, Technology and Research (A*STAR), Singapore.; c Department of Immunology and Infectious Diseases, Harvard TH Chan School of Public Health, Boston, USA.; d Antigen Discovery Inc., Irvine, USA.

## Abstract

**Objective:**

To analyse the proportions of protein identity between Zika virus and dengue, Japanese encephalitis, yellow fever, West Nile and chikungunya viruses as well as polymorphism between different Zika virus strains.

**Methods:**

We used published protein sequences for the Zika virus and obtained protein sequences for the other viruses from the National Center for Biotechnology Information (NCBI) protein database or the NCBI virus variation resource. We used BLASTP to find regions of identity between viruses. We quantified the identity between the Zika virus and each of the other viruses, as well as within-Zika virus polymorphism for all amino acid *k*-mers across the proteome, with *k* ranging from 6 to 100. We assessed accessibility of protein fragments by calculating the solvent accessible surface area for the envelope and nonstructural-1 (NS1) proteins.

**Findings:**

In total, we identified 294 Zika virus protein fragments with both low proportion of identity with other viruses and low levels of polymorphisms among Zika virus strains. The list includes protein fragments from all Zika virus proteins, except NS3. NS4A has the highest number (190 *k*-mers) of protein fragments on the list.

**Conclusion:**

We provide a candidate list of protein fragments that could be used when developing a sensitive and specific serological test to detect previous Zika virus infections.

## Résumé

**Objectif:**

Analyser les pourcentages de similarité protéique entre le virus Zika et les virus de la dengue, de l'encéphalite japonaise, de la fièvre jaune, du Nil occidental et du chikungunya, ainsi que le polymorphisme entre différentes souches du virus Zika.

**Méthodes:**

Nous avons utilisé les séquences protéiques publiées du virus Zika et avons obtenu les séquences protéiques des autres virus dans la banque protéique du National Center for Biotechnology Information (NCBI) ou dans la base de données Virus Variation du NCBI. Nous avons utilisé BLASTP pour identifier les régions de similarité entre les virus. Nous avons quantifié la similarité entre le virus Zika et chacun des autres virus ainsi que le polymorphisme du virus Zika pour tous les *k*-mers d'acides aminés, dans tout le protéome, avec *k* allant de 6 à 100. Nous avons étudié l'accessibilité des fragments protéiques en calculant la surface accessible au solvant pour les protéines d'enveloppe et non structurale-1 (NS1).

**Résultats:**

Au total, nous avons identifié 294 fragments protéiques du virus Zika qui présentent à la fois un faible degré de similarité avec les autres virus et un faible degré de polymorphisme entre les souches du virus Zika. Notre liste comprend des fragments protéiques issus de toutes les protéines du virus Zika, à l'exception de la protéine NS3. Le plus grand nombre de fragments protéiques de notre liste (190 *k*-mers) correspond à la protéine NS4A.

**Conclusion:**

Nous proposons une liste de fragments protéiques candidats, qui pourraient être utilisés pour concevoir un test sérologique sensible et spécifique pour dépister les infections antérieures par le virus Zika.

## Resumen

**Objective:**

Analizar las proporciones de identidad proteica entre el virus de Zika y los virus del dengue, la encefalitis japonesa, la fiebre amarilla, el Nilo Occidental y el chikungunya, así como el polimorfismo entre las distintas cepas del virus de Zika.

**Métodos:**

Se utilizaron secuencias de proteínas publicadas para el virus de Zika y secuencias de proteínas obtenidas para los otros virus de la base de datos de proteínas del Centro Nacional para la Información Biotecnológica (NCBI) o la fuente de información sobre la variación de virus del NCBI. Se utilizó el programa BLASTP para encontrar regiones de identidad entre los virus. Se cuantificó la identidad entre el virus de Zika y cada uno de los otros virus, así como el polimorfismo del virus de Zika para todos los *k*-mers de aminoácidos a través del proteoma, con una variación de *k* de 6 a 100. Se evaluó la accesibilidad de los fragmentos proteicos calculando la superficie accesible solvente para las proteínas de envoltura y no estructurales 1 (NS1).

**Resultados:**

En total, se identificaron 294 fragmentos proteicos del virus de Zika con una proporción escasa de identidad con otros virus y con niveles bajos de polimorfismos entre las distintas cepas del virus de Zika. En la lista se incluyen fragmentos proteicos de todas las proteínas del virus de Zika, salvo la NS3. La NS4A cuenta con el mayor número (190 *k*-mers) de fragmentos proteicos de la lista.

**Conclusión:**

Se proporcionó una lista de posibles fragmentos proteicos que podrían utilizarse para desarrollar una prueba serológica sensible y específica para detectar infecciones del virus de Zika anteriores.

## ملخص

**الغرض:**

تحليل مقدار تماثل البروتين بين فيروس زيكا وحمى الضنك، والتهاب الدماغ الياباني، وحمى الصفراء، وفيروس غرب النيل، وشيكونغونيا فضلاً عن تعددية الأشكال بين سلالات فيروس زيكا.

**الطريقة:**

استخدمنا تسلسلات البروتين المنشورة لفيروس زيكا وحصلنا على تسلسلات البروتين لغيره من الفيروسات من قاعدة البيانات الخاصة بالبروتين للمركز الوطني لمعلومات التقانة الحيوية (NCBI) أو مورد اختلاف الفيروسات لمركز NCBI. كما استخدمنا أداة BLASTP للعثور على مناطق التماثل بين الفيروسات. وقمنا بإجراء تحديد كمي لمقدار التماثل بين فيروس زيكا وكلٍ من الفيروسات الأخرى فضلاً عن تعددية الأشكال في فيروس زيكا نفسه لكل ميرات *k * للأحماض الأمينية السائدة عبر البروتيوم، حيث تتراوح *k * من 6 إلى 100. وقمنا بتقييم إمكانية الوصول إلى شظايا البروتين من خلال حساب المساحة السطحية التي يمكن للمذيبات الوصول من خلالها للبروتينات المغلفة والبروتين اللابنيوي-1 (NS1).

**النتائج:**

إجمالاً، قمنا بتحديد 294 شظية من شظايا البروتين الخاص بفيروس زيكا مع انخفاض نسبة التماثل مع الفيروسات الأخرى وانخفاض مستويات تعدد الأشكال بين سلالات فيروس زيكا. وتتضمن القائمة شظايا البروتين من جميع بروتينات فيروس زيكا باستثناء بروتين NS3. ويتمتع بروتين NS4A بالرقم الأكبر (190 من ميرات *k *) من شظايا البروتين الواردة في القائمة.

**الاستنتاج:**

قمنا بإنشاء قائمة مرشحين لشظايا البروتين والتي يمكن استخدامها عند تطوير اختبار مصلي حساس ومحدد لاكتشاف الحالات السابقة للإصابة بفيروس زيكا.

## 摘要

**目的:**

旨在分析寨卡病毒、登革热、流行性乙型脑炎、黄热病、西尼罗河以及基孔肯雅热病毒之间的蛋白质识别率以及不同寨卡病毒株之间的多态性。

**方法:**

我们使用已公布的寨卡病毒蛋白质序列，并从国家生物技术信息中心 (NCBI) 蛋白质数据库或国家生物技术信息中心 (NCBI) 病毒变异资源中获取了其他病毒的蛋白质序列。 我们使用 BLASTP 来找出病毒之间的识别区域。 我们量化了寨卡病毒和其他各种病毒之间的蛋白质识别以及寨卡病毒内部多态性，以识别蛋白质组中的所有氨基酸 *k*-mer，其中 *k* 的变化范围为 6 到 100。通过计算外膜蛋白和非结构蛋白 1 (NS1) 的溶剂可及表面，我们对蛋白质片段的可及性进行了评估。

**结果:**

我们共识别出 294 个寨卡病毒蛋白质片段，相较于其他病毒，其识别率较低，且寨卡病毒株之间的多态性程度较低。 上述清单包括所有寨卡病毒蛋白质的蛋白质片段，非结构蛋白 3 (NS3) 除外。 清单中，非结构蛋白 4A (NS4A) 的蛋白质片段数目（190 个 *k*-mer）最高。

**结论:**

我们提供了一份蛋白质片段补充目录，可在开发敏感的特殊血清学测试时使用，以检测之前的寨卡病毒感染情况。

## Резюме

**Цель:**

Проанализировать пропорции белковой идентичности между вирусом Зика и вирусами лихорадки денге, японского энцефалита, желтой лихорадки, лихорадки Западного Нила и лихорадки чикунгунья, а также полиморфизм между различными штаммами вируса Зика.

**Методы:**

Мы использовали опубликованные последовательности белка для вируса Зика и получили последовательности белка для других вирусов из базы данных Национального центра биотехнологической информации (NCBI) или ресурса вирусных вариаций NCBI. Мы использовали программу BLASTP, чтобы найти области идентичности между вирусами. Мы провели количественную оценку идентичности между вирусом Зика и каждым из других вирусов, а также оценку полиморфизма между различными штаммами вируса Зика для всех *k*-меров аминокислот всего протеома, где *k* находится в пределах от 6 до 100. Мы оценили доступности фрагментов белка путем расчета доступной для растворителя области поверхности для белков оболочки и неструктурного белка-1 (NS1).

**Результаты:**

В целом мы идентифицировали 294 фрагмента белка вируса Зика с низкой долей идентичности с другими вирусами и низкими уровнями полиморфизма среди штаммов вируса Зика. Этот список включает белковые фрагменты от всех белков вируса Зика, за исключением NS3. В этом списке NS4A имеет самое большое количество (190 *k*-меров) фрагментов белка.

**Вывод:**

Мы подготовили список белковых фрагментов-кандидатов, которые можно использовать при разработке чувствительного и специфического серологического теста для выявления ранее обнаруженных инфекций, вызываемых вирусом Зика.

## Introduction

Monitoring the geographic and the demographic distribution of people infected with Zika virus is important for informing decision-makers and researchers during the ongoing epidemic. Health officials also need further knowledge about the associations between Zika virus infection and its sequelae, such as microcephaly and Guillain–Barré syndrome. However, the absence of a sensitive and specific serological test for detecting prior Zika virus infection impedes research. According to the World Health Organization’s *Target product profiles for better diagnostic tests for Zika virus infection*,^1^ such a test must be able to differentiate between chikungunya, dengue and Zika viruses, since these mosquito-borne arboviruses can be co-circulating and can cause similar symptoms.^2^

Dengue and Zika viruses belong to the virus family *Flaviviridae*, while chikungunya virus belongs to the *Togaviridae* family. Although they belong to different virus families, Zika and chikungunya viruses share some similarities in envelope protein folding and membrane fusion mechanisms.^3^

Active Zika virus infections can be detected by nucleic acid-based diagnostic tools.^4^^,^^5^ However, developing serological diagnostic tests to detect previous Zika virus infections has been challenging, because of cross-reactivity between antibodies against different arboviruses.^6^^–^^12^ Hence, current serological assays, such as enzyme-linked immunosorbent assay (ELISA) and plaque reduction neutralization tests, may not be able to distinguish if a person has been infected with Zika virus or another flavivirus or if a person has received a previous yellow fever or Japanese encephalitis vaccination.^13^^,^^14^ A study has shown that neutralizing monoclonal antibodies generated against recombinant fragments of the envelope protein of dengue virus serotype 2 tend to be cross-reactive among flaviviruses, while nonneutralizing antibodies seem to be virus specific.^15^

We hypothesize that immunogenic protein regions with sequence dissimilarity may exist across arthropod-borne viruses (arboviruses) and that antibodies targeting these regions may be less likely to be cross-reactive. Identifying such regions could aid the development of specific microarray-based serological tests, such as a peptide microarray, to detect Zika virus and/or other related viruses. A peptide microarray is a high-throughput method for detecting interactions between peptides and antibodies and is composed of multiple spots of peptides on a solid surface.^16^ We also hypothesize that protein regions that are more conserved among different strains of the Zika virus are more likely to contribute to the sensitivity of the peptide microarray. Thus, to identify Zika virus conserved protein fragments that are variable among other virus species, we analysed proportions of protein sequence identity across virus species and protein polymorphism among different strains of Zika virus. We analysed the flaviviruses Zika, dengue, West Nile, Japanese encephalitis and yellow fever, and the alphavirus chikungunya.

## Methods

We used publicly available proteomic sequencing data (Table 1). For the Zika virus, we used data set A from Faria et al.^17^ We downloaded the protein sequences of Japanese encephalitis virus, yellow fever virus and chikungunya virus from the National Center for Biotechnology Information (NCBI) protein database and the sequences for dengue virus serotypes 1–4 and West Nile virus from NCBI virus variation resource.^18^

**Table 1:** Proteomic sequencing data used to compare identity between viruses and within viruses

Species	Collection date	WHO Region	No. of samples
ZIKV	1947–2015	African, Americas, Western Pacific	34
DENV1	01/01/2010– 06/01/2016	African, Americas, European, South-East Asia, Western Pacific	171
DENV2	01/01/2010– 06/01/2016	Americas, Eastern Mediterranean, South-East Asia, Western Pacific	158
DENV3	01/01/2010– 06/01/2016	Americas, Eastern Mediterranean, South-East Asia, Western Pacific	62
DENV4	01/01/2010– 06/01/2016	Americas, South-East Asia, Western Pacific	58
WNV	01/01/2008–06/01/2016	Americas, European, South-East Asia,	44
JEV	1951–2012	South-East Asia, Western Pacific	19
YFV	1981–2016	African, Americas, Western Pacific	31
CHIKV	1953-2015	African, Americas, European, South-East Asia, Western Pacific	212

CHIKV: chikungunya virus; DENV1−4: dengue virus serotype 1; JEV: Japanese encephalitis virus; WHO: World Health Organization; WNV: West Nile virus; YFV; yellow fever virus; ZIKV: Zika virus.

Note: For ZIKV, we used data set A from Faria et al.^17^ We downloaded the protein sequences of JEV, YFV and CHIKV from the National Center of Biotechnology Information (NCBI) protein database and sequences for DENV serotypes 1–4 and WNV from NCBI virus variation resource.^18^

We used BLASTP^19^ to find regions of identity between arboviruses, applying a default Expect (*E)*-value threshold of 10, that is the expected number of hits of the observed similarity, by chance, is fewer than 10. The results are robust and we obtained the same results when *E*-value thresholds were 5 or 50. When comparing the chikungunya and the Zika viruses, we used an *E*-value threshold of 1000, because chikungunya does not belong to the *Flaviviridae* family and we could not identify any regions of similarity when using an *E*-value threshold of 10. For all protein fragments across the proteome, we calculated the proportion of shared amino acids between virus species and polymorphism among different Zika virus strains. We analysed protein fragments of different lengths, so called *k*-mers (where *k* is the amino acid length of the protein fragment), with *k* equal to 6 or ranging from 10 to 100. We used a sliding window approach, where we moved the window one amino acid at a time along the proteome to include every possible *k*-mer. To be conservative, we identified protein fragment identity between species by the maximum identity among all the pairs of strains for each window considered. For analysing the identity with dengue virus, we used the highest identity between the Zika virus and all four serotypes of the dengue virus for each window considered. To assess if protein identity between the Zika virus and each of the dengue serotype was significantly associated with polymorphism within each dengue virus serotype, we calculated *P*-values by using Pearson's correlation test.

To identify polymorphisms within viruses, we used both the average pairwise difference and the proportion of polymorphic sites. Average pairwise difference is calculated by averaging the proportions of differences in peptide sequences from all pairs of the virus strains. We chose to plot the proportion of polymorphic sites in the figures because it is less sensitive to population structure and/or sampling bias.

To identify potential protein fragments that could be used for diagnostic tests, we selected *k*-mers with low proportion of identity between the Zika virus and other arboviruses as well as low polymorphism between different strains of Zika virus as lead candidate protein fragments. The rationale for this approach was that fragments with low between-species identity and low within-species polymorphism are most likely to have both the required specificity and sensitivity for such tests. We chose *k*-mers in the bottom quintile of values of identity and polymorphism for each *k*-mer length.

Insights into protein structures are critical for assessing the possible antigenicity of peptides, because buried peptides are less likely to be antigenic.^20^ To determine if any of the fragments are exposed or buried in the two Zika virus proteins with available protein structures, the envelope protein and the non-structural (NS) protein 1, we calculated the solvent accessible surface area for each amino acid. We used the published structures of dimeric NS1 (protein data bank identification, PDB ID: 5GS6)^21^ and the envelope protein in the biological assembly of the mature virus (PDB ID: 5IRE).^22^ To calculate the solvent accessible surface area, we used the linear combinations of pairwise overlaps method^23^ and used 10 Å^2^ as the upper limit for buried residues, as this value corresponds to half the surface area of a single water molecule. The regions at the *C*-terminal end of the dengue virus envelope protein interact with the viral lipid membrane^24^ and are unlikely to be exposed. Due to the high structural similarity of the envelope proteins between dengue and Zika viruses, we assume that the region from residue 404 to the *C*-terminus in Zika virus envelope protein is also buried. For the lead candidate list, we excluded the *k*-mers without any continuous exposed peptides longer than five amino acids in the two proteins, because exposed peptides are more likely to be antigenic. The threshold of five amino acids was chosen because 99.7% of experimentally determined antigenic B-cell epitopes for flaviviruses found in Virus Pathogen Database and Analysis Resource database are longer than five amino acids.^25^ We obtained the list of theses epitopes through the database’s web site at http://www.viprbrc.org/.

## Results

**Fig. 1:**
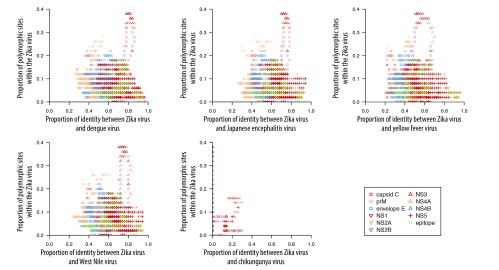
Zika virus polymorphism versus identity between Zika virus and other arboviruses, 50-mers across the Zika virus proteome

**Fig. 2:**
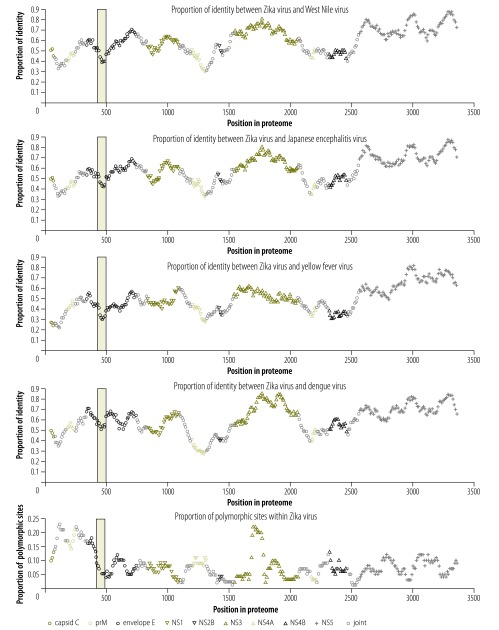
Sliding-window identity between Zika virus and other flaviviruses and within-Zika virus polymorphism

On average, Zika virus shares 55.6% amino acid sequence identity with dengue virus, 46.0% with yellow fever virus, 56.1% with Japanese encephalitis virus, 57.0% with West Nile virus and 1.3% with chikungunya virus. The identity between Zika virus and other viruses and Zika virus polymorphism for all *k*-mers are available from the corresponding author. As an example, Fig. 1 and Fig. 2 show the identity between Zika virus and other viruses investigated and polymorphisms within the Zika virus for all 50-mer peptides.

E: envelope; NS; non-structural; prM; precursor membrane.

Notes: 50-mers across the Zika virus proteome were analysed, using a sliding window approach. Fifty-mers containing known epitopes in non-Zika virus flaviviruses are shown in green. Polymorphic sites are the sites that vary among different strains of Zika virus.

E: envelope; NS; non-structural; prM; precursor membrane.

Notes: The window size is 50 amino acids and step size is 5 amino acids. The light green shaded area (position 430–500) shows low between-species identity and low within-Zika virus polymorphism.

**Fig. 3:**
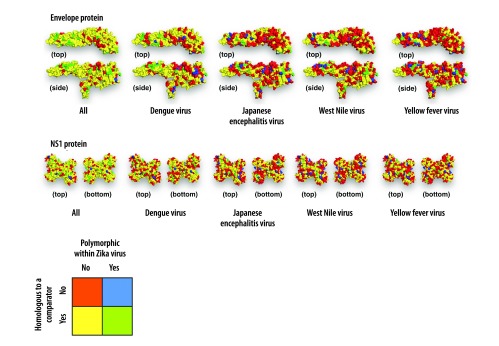
Mapping per-site identity and polymorphism onto the structures of Zika virus envelope protein and nonstructural protein 1 dimer

Fig. 3 shows protein fragments mapped to the corresponding envelope or NS1 proteins. The exposed areas of the proteins show regions with both low identity with other flaviviruses and low Zika virus polymorphism.

NS1: nonstructural protein 1.

Notes: Sites polymorphic within Zika virus or with high identity between Zika virus and other viruses could compromise respectively sensitivity and specificity of a serological test. Thus the most useful sites are expected to be those that lack identity with other viruses and lack polymorphism within Zika virus, shown in red. Identity between a comparator virus and Zika virus is shown in yellow; polymorphism within Zika virus is shown in blue; and sites with both properties are shown in green. The side view of the envelope protein shows the two transmembrane helices on the bottom. These helices likely remain buried within the lipid bilayer envelope and hence are unavailable for interactions with antibodies. In the structures including all viruses, sites are shown as homologous if they are homologous between Zika virus and any of the flaviviruses. Homologous regions are larger for dengue virus because sites are shown as homologous if they are homologous between Zika virus and any of the four dengue virus serotypes.

The lead candidate list for developing a specific and sensitive microarray-based serological test contains 294 protein fragments. These fragments have low similarity between viruses, low polymorphism within the Zika virus and continuous exposed peptides longer than five amino acids (Table 2; available at: http://www.who.int/bulletin/volumes/95/7/16-182105). The list excluded 10.9% (36/330) of *k*-mers containing previously identified B-cell epitopes for other flaviviruses than Zika, because they are likely to be cross-reactive. Protein fragments from all Zika virus proteins, except NS3, are present in the list. NS4A has the highest number (190 *k*-mers) of candidate protein fragments (Table 3).

**Table 2:** The lead candidate list of Zika virus protein fragments with low proportion of identity with other flaviviruses and low polymorphism

Position in proteome, aa		Protein	Average pairwise difference	Polymorphic sites, %	*k*-mer^a^	Homology with other flaviviruses, %				Peptide sequence
Start	End					DENV	JEV	YFV	WNV	
26	95	capsid C	0.0078	0.0429	70	0.4857	0.4857	0.2286	0.5000	PFGGLKRLPAGLLLGHGPIRMVLAILAFLRFTAIKPSLGLINRWGSVGKKEAMEIIKKFKKDLAAMLRII
28	97	capsid C	0.0019	0.0286	70	0.5000	0.4714	0.2286	0.5000	GGLKRLPAGLLLGHGPIRMVLAILAFLRFTAIKPSLGLINRWGSVGKKEAMEIIKKFKKDLAAMLRIINA
32	101	capsid C	0.0077	0.0429	70	0.5000	0.4714	0.2571	0.4857	RLPAGLLLGHGPIRMVLAILAFLRFTAIKPSLGLINRWGSVGKKEAMEIIKKFKKDLAAMLRIINARKEK
33	102	capsid C	0.0077	0.0429	70	0.4857	0.4714	0.2571	0.4857	LPAGLLLGHGPIRMVLAILAFLRFTAIKPSLGLINRWGSVGKKEAMEIIKKFKKDLAAMLRIINARKEKK
34	103	capsid C	0.0077	0.0429	70	0.4857	0.4857	0.2571	0.5000	PAGLLLGHGPIRMVLAILAFLRFTAIKPSLGLINRWGSVGKKEAMEIIKKFKKDLAAMLRIINARKEKKR
35	104	capsid C	0.0077	0.0429	70	0.4857	0.4857	0.2571	0.5000	AGLLLGHGPIRMVLAILAFLRFTAIKPSLGLINRWGSVGKKEAMEIIKKFKKDLAAMLRIINARKEKKRR
45	94	capsid C	0.0027	0.0400	50	0.4800	0.4600	0.2600	0.4800	RMVLAILAFLRFTAIKPSLGLINRWGSVGKKEAMEIIKKFKKDLAAMLRI
54	93	capsid C	0.0033	0.0500	40	0.4750	0.4000	0.2500	0.4250	LRFTAIKPSLGLINRWGSVGKKEAMEIIKKFKKDLAAMLR
55	94	capsid C	0.0033	0.0500	40	0.4750	0.4000	0.2500	0.4250	RFTAIKPSLGLINRWGSVGKKEAMEIIKKFKKDLAAMLRI
56	95	capsid C	0.0033	0.0500	40	0.4750	0.4000	0.2500	0.4250	FTAIKPSLGLINRWGSVGKKEAMEIIKKFKKDLAAMLRII
57	96	capsid C	0.0033	0.0500	40	0.4750	0.4000	0.2250	0.4250	TAIKPSLGLINRWGSVGKKEAMEIIKKFKKDLAAMLRIIN
58	97	capsid C	0.0033	0.0500	40	0.4750	0.3750	0.2000	0.4000	AIKPSLGLINRWGSVGKKEAMEIIKKFKKDLAAMLRIINA
59	98	capsid C	0.0033	0.0500	40	0.4750	0.3750	0.2250	0.4000	IKPSLGLINRWGSVGKKEAMEIIKKFKKDLAAMLRIINAR
60	99	capsid C	0.0033	0.0500	40	0.4750	0.4000	0.2500	0.3750	KPSLGLINRWGSVGKKEAMEIIKKFKKDLAAMLRIINARK
61	100	capsid C	0.0033	0.0500	40	0.4750	0.4000	0.2250	0.3750	PSLGLINRWGSVGKKEAMEIIKKFKKDLAAMLRIINARKE
87	92	capsid C	0.0000	0.0000	6	0.3333	0.1667	0.1667	0.1667	DLAAML
131	136	pr	0.0000	0.0000	6	0.1667	0.1667	0.0000	0.1667	AYYMYL
132	137	pr	0.0000	0.0000	6	0.1667	0.1667	0.0000	0.1667	YYMYLD
133	138	pr	0.0000	0.0000	6	0.1667	0.1667	0.0000	0.1667	YMYLDR
231	240	membrane	0.0000	0.0000	10	0.3000	0.3000	0.2000	0.3000	SQTWLESREY
411	450	envelope	0.0044	0.0750	40	0.4500	0.4250	0.2250	0.3750	CSKKMTGKSIQPENLEYRIMLSVHGSQHSGMIGHETDENR
412	451	envelope	0.0044	0.0750	40	0.4250	0.4250	0.2000	0.3500	SKKMTGKSIQPENLEYRIMLSVHGSQHSGMIGHETDENRA
413	452	envelope	0.0044	0.0750	40	0.4250	0.4250	0.2250	0.3750	KKMTGKSIQPENLEYRIMLSVHGSQHSGMIGHETDENRAK
414	453	envelope	0.0044	0.0750	40	0.4250	0.4250	0.2000	0.3750	KMTGKSIQPENLEYRIMLSVHGSQHSGMIGHETDENRAKV
415	454	envelope	0.0044	0.0750	40	0.4500	0.4000	0.2000	0.3500	MTGKSIQPENLEYRIMLSVHGSQHSGMIGHETDENRAKVE
419	448	envelope	0.0020	0.0333	30	0.4667	0.4000	0.2000	0.3667	SIQPENLEYRIMLSVHGSQHSGMIGHETDE
420	449	envelope	0.0020	0.0333	30	0.4667	0.4000	0.2000	0.3667	IQPENLEYRIMLSVHGSQHSGMIGHETDEN
421	450	envelope	0.0020	0.0333	30	0.4667	0.3667	0.2000	0.3333	QPENLEYRIMLSVHGSQHSGMIGHETDENR
422	451	envelope	0.0020	0.0333	30	0.4667	0.3667	0.2000	0.3000	PENLEYRIMLSVHGSQHSGMIGHETDENRA
436	445	envelope	0.0000	0.0000	10	0.4000	0.3000	0.2000	0.3000	SQHSGMIGHE
438	447	envelope	0.0000	0.0000	10	0.4000	0.3000	0.2000	0.3000	HSGMIGHETD
439	448	envelope	0.0000	0.0000	10	0.4000	0.3000	0.2000	0.3000	SGMIGHETDE
440	449	envelope	0.0000	0.0000	10	0.4000	0.2000	0.2000	0.2000	GMIGHETDEN
441	450	envelope	0.0000	0.0000	10	0.4000	0.2000	0.2000	0.2000	MIGHETDENR
442	451	envelope	0.0000	0.0000	10	0.4000	0.3000	0.2000	0.2000	IGHETDENRA
626	665	envelope	0.0029	0.0500	40	0.4000	0.4750	0.3000	0.4750	KVPAQMAVDMQTLTPVGRLITANPVITESTENSKMMLELD
627	666	envelope	0.0029	0.0500	40	0.4000	0.4750	0.3000	0.4750	VPAQMAVDMQTLTPVGRLITANPVITESTENSKMMLELDP
629	658	envelope	0.0020	0.0333	30	0.4333	0.4667	0.3000	0.4333	AQMAVDMQTLTPVGRLITANPVITESTENS
913	918	NS1	0.0000	0.0000	6	0.3333	0.0000	0.0000	0.0000	FVRAAK
913	922	NS1	0.0000	0.0000	10	0.4000	0.1000	0.2000	0.2000	FVRAAKTNNS
914	919	NS1	0.0000	0.0000	6	0.3333	0.0000	0.0000	0.0000	VRAAKT
915	920	NS1	0.0000	0.0000	6	0.3333	0.1667	0.1667	0.1667	RAAKTN
1294	1343	NS2A	0.0043	0.0600	50	0.3600	0.3600	0.3000	0.3200	LAILAALTPLARGTLLVAWRAGLATCGGFMLLSLKGKGSVKKNLPFVMAL
1295	1344	NS2A	0.0043	0.0600	50	0.3800	0.3600	0.2800	0.3200	AILAALTPLARGTLLVAWRAGLATCGGFMLLSLKGKGSVKKNLPFVMALG
1299	1318	NS2A	0.0000	0.0000	20	0.3500	0.3500	0.2500	0.3500	ALTPLARGTLLVAWRAGLAT
1299	1338	NS2A	0.0018	0.0250	40	0.3500	0.3250	0.2500	0.3250	ALTPLARGTLLVAWRAGLATCGGFMLLSLKGKGSVKKNLP
1299	1348	NS2A	0.0043	0.0600	50	0.3600	0.3400	0.3000	0.3000	ALTPLARGTLLVAWRAGLATCGGFMLLSLKGKGSVKKNLPFVMALGLTAV
1300	1319	NS2A	0.0000	0.0000	20	0.3500	0.3500	0.3000	0.3500	LTPLARGTLLVAWRAGLATC
1300	1339	NS2A	0.0018	0.0250	40	0.3500	0.3250	0.2500	0.3250	LTPLARGTLLVAWRAGLATCGGFMLLSLKGKGSVKKNLPF
1300	1349	NS2A	0.0043	0.0600	50	0.3600	0.3400	0.3000	0.3000	LTPLARGTLLVAWRAGLATCGGFMLLSLKGKGSVKKNLPFVMALGLTAVR
1301	1320	NS2A	0.0000	0.0000	20	0.3500	0.3500	0.2500	0.3500	TPLARGTLLVAWRAGLATCG
1301	1340	NS2A	0.0018	0.0250	40	0.3750	0.3000	0.2500	0.3000	TPLARGTLLVAWRAGLATCGGFMLLSLKGKGSVKKNLPFV
1302	1311	NS2A	0.0000	0.0000	10	0.4000	0.3000	0.2000	0.3000	PLARGTLLVA
1302	1321	NS2A	0.0000	0.0000	20	0.3500	0.3000	0.2000	0.3000	PLARGTLLVAWRAGLATCGG
1303	1322	NS2A	0.0000	0.0000	20	0.3500	0.2500	0.1500	0.2500	LARGTLLVAWRAGLATCGGF
1304	1323	NS2A	0.0000	0.0000	20	0.3500	0.2500	0.1500	0.2500	ARGTLLVAWRAGLATCGGFM
1305	1324	NS2A	0.0000	0.0000	20	0.4000	0.3000	0.1500	0.3000	RGTLLVAWRAGLATCGGFML
1309	1318	NS2A	0.0000	0.0000	10	0.4000	0.3000	0.2000	0.3000	LVAWRAGLAT
1310	1319	NS2A	0.0000	0.0000	10	0.4000	0.2000	0.2000	0.2000	VAWRAGLATC
1311	1320	NS2A	0.0000	0.0000	10	0.4000	0.3000	0.2000	0.3000	AWRAGLATCG
1312	1321	NS2A	0.0000	0.0000	10	0.3000	0.3000	0.2000	0.3000	WRAGLATCGG
1313	1322	NS2A	0.0000	0.0000	10	0.2000	0.3000	0.2000	0.3000	RAGLATCGGF
1314	1323	NS2A	0.0000	0.0000	10	0.1000	0.2000	0.2000	0.2000	AGLATCGGFM
1315	1324	NS2A	0.0000	0.0000	10	0.2000	0.3000	0.2000	0.3000	GLATCGGFML
1317	1322	NS2A	0.0000	0.0000	6	0.1667	0.1667	0.1667	0.1667	ATCGGF
1318	1323	NS2A	0.0000	0.0000	6	0.1667	0.1667	0.1667	0.1667	TCGGFM
1326	1331	NS2A	0.0000	0.0000	6	0.1667	0.1667	0.1667	0.1667	SLKGKG
1328	1333	NS2A	0.0000	0.0000	6	0.1667	0.1667	0.1667	0.1667	KGKGSV
1328	1337	NS2A	0.0000	0.0000	10	0.3000	0.3000	0.2000	0.3000	KGKGSVKKNL
1354	1363	NS2A	0.0000	0.0000	10	0.3000	0.3000	0.2000	0.3000	INVVGLLLLT
1459	1468	NS2B	0.0000	0.0000	10	0.3000	0.3000	0.2000	0.3000	GPPMREIILK
1460	1469	NS2B	0.0000	0.0000	10	0.3000	0.2000	0.2000	0.2000	PPMREIILKV
1461	1470	NS2B	0.0000	0.0000	10	0.3000	0.2000	0.2000	0.3000	PMREIILKVV
1462	1467	NS2B	0.0000	0.0000	6	0.3333	0.1667	0.1667	0.1667	MREIIL
1462	1471	NS2B	0.0000	0.0000	10	0.4000	0.1000	0.1000	0.2000	MREIILKVVL
1463	1472	NS2B	0.0000	0.0000	10	0.4000	0.1000	0.1000	0.2000	REIILKVVLM
1474	1493	NS2B	0.0000	0.0000	20	0.4000	0.2500	0.2000	0.3500	ICGMNPIAIPFAAGAWYVYV
1475	1494	NS2B	0.0000	0.0000	20	0.4000	0.3000	0.2500	0.3000	CGMNPIAIPFAAGAWYVYVK
1476	1495	NS2B	0.0000	0.0000	20	0.4000	0.3500	0.2500	0.3000	GMNPIAIPFAAGAWYVYVKT
1477	1496	NS2B	0.0000	0.0000	20	0.3500	0.3500	0.2500	0.3000	MNPIAIPFAAGAWYVYVKTG
1478	1497	NS2B	0.0000	0.0000	20	0.3500	0.4000	0.2500	0.3500	NPIAIPFAAGAWYVYVKTGK
1483	1492	NS2B	0.0000	0.0000	10	0.4000	0.2000	0.2000	0.3000	PFAAGAWYVY
1484	1493	NS2B	0.0000	0.0000	10	0.3000	0.2000	0.2000	0.2000	FAAGAWYVYV
2116	2215	NS4A	0.0091	0.0600	100	0.3900	0.3500	0.3500	0.4400	GAAFGVMEALGTLPGHMTERFQEAIDNLAVLMRAETGSRPYKAAAAQLPETLETIMLLGLLGTVSLGIFFVLMRNKGIGKMGFGMVTLGASAWLMWLSEI
2117	2216	NS4A	0.0091	0.0600	100	0.3900	0.3500	0.3400	0.4400	AAFGVMEALGTLPGHMTERFQEAIDNLAVLMRAETGSRPYKAAAAQLPETLETIMLLGLLGTVSLGIFFVLMRNKGIGKMGFGMVTLGASAWLMWLSEIE
2118	2217	NS4A	0.0084	0.0500	100	0.4000	0.3500	0.3400	0.4400	AFGVMEALGTLPGHMTERFQEAIDNLAVLMRAETGSRPYKAAAAQLPETLETIMLLGLLGTVSLGIFFVLMRNKGIGKMGFGMVTLGASAWLMWLSEIEP
2119	2218	NS4A	0.0084	0.0500	100	0.4000	0.3400	0.3300	0.4400	FGVMEALGTLPGHMTERFQEAIDNLAVLMRAETGSRPYKAAAAQLPETLETIMLLGLLGTVSLGIFFVLMRNKGIGKMGFGMVTLGASAWLMWLSEIEPA
2120	2179	NS4A	0.0057	0.0500	60	0.4333	0.3333	0.3500	0.3833	GVMEALGTLPGHMTERFQEAIDNLAVLMRAETGSRPYKAAAAQLPETLETIMLLGLLGTV
2120	2189	NS4A	0.0049	0.0429	70	0.3857	0.3286	0.3286	0.4286	GVMEALGTLPGHMTERFQEAIDNLAVLMRAETGSRPYKAAAAQLPETLETIMLLGLLGTVSLGIFFVLMR
2120	2199	NS4A	0.0052	0.0500	80	0.4125	0.3875	0.3375	0.4625	GVMEALGTLPGHMTERFQEAIDNLAVLMRAETGSRPYKAAAAQLPETLETIMLLGLLGTVSLGIFFVLMRNKGIGKMGFG
2120	2209	NS4A	0.0046	0.0444	90	0.3889	0.3556	0.3333	0.4667	GVMEALGTLPGHMTERFQEAIDNLAVLMRAETGSRPYKAAAAQLPETLETIMLLGLLGTVSLGIFFVLMRNKGIGKMGFGMVTLGASAWL
2120	2219	NS4A	0.0041	0.0400	100	0.3900	0.3400	0.3300	0.4400	GVMEALGTLPGHMTERFQEAIDNLAVLMRAETGSRPYKAAAAQLPETLETIMLLGLLGTVSLGIFFVLMRNKGIGKMGFGMVTLGASAWLMWLSEIEPAR
2121	2180	NS4A	0.0057	0.0500	60	0.4333	0.3333	0.3500	0.3833	VMEALGTLPGHMTERFQEAIDNLAVLMRAETGSRPYKAAAAQLPETLETIMLLGLLGTVS
2121	2190	NS4A	0.0049	0.0429	70	0.3857	0.3286	0.3286	0.4143	VMEALGTLPGHMTERFQEAIDNLAVLMRAETGSRPYKAAAAQLPETLETIMLLGLLGTVSLGIFFVLMRN
2121	2200	NS4A	0.0052	0.0500	80	0.4125	0.3875	0.3500	0.4500	VMEALGTLPGHMTERFQEAIDNLAVLMRAETGSRPYKAAAAQLPETLETIMLLGLLGTVSLGIFFVLMRNKGIGKMGFGM
2121	2210	NS4A	0.0046	0.0444	90	0.3889	0.3556	0.3444	0.4556	VMEALGTLPGHMTERFQEAIDNLAVLMRAETGSRPYKAAAAQLPETLETIMLLGLLGTVSLGIFFVLMRNKGIGKMGFGMVTLGASAWLM
2121	2220	NS4A	0.0041	0.0400	100	0.4000	0.3500	0.3400	0.4400	VMEALGTLPGHMTERFQEAIDNLAVLMRAETGSRPYKAAAAQLPETLETIMLLGLLGTVSLGIFFVLMRNKGIGKMGFGMVTLGASAWLMWLSEIEPARI
2122	2181	NS4A	0.0057	0.0500	60	0.4333	0.3333	0.3500	0.4000	MEALGTLPGHMTERFQEAIDNLAVLMRAETGSRPYKAAAAQLPETLETIMLLGLLGTVSL
2122	2191	NS4A	0.0049	0.0429	70	0.4000	0.3429	0.3429	0.4286	MEALGTLPGHMTERFQEAIDNLAVLMRAETGSRPYKAAAAQLPETLETIMLLGLLGTVSLGIFFVLMRNK
2122	2201	NS4A	0.0052	0.0500	80	0.4125	0.3875	0.3500	0.4625	MEALGTLPGHMTERFQEAIDNLAVLMRAETGSRPYKAAAAQLPETLETIMLLGLLGTVSLGIFFVLMRNKGIGKMGFGMV
2122	2211	NS4A	0.0046	0.0444	90	0.4000	0.3667	0.3444	0.4667	MEALGTLPGHMTERFQEAIDNLAVLMRAETGSRPYKAAAAQLPETLETIMLLGLLGTVSLGIFFVLMRNKGIGKMGFGMVTLGASAWLMW
2122	2221	NS4A	0.0041	0.0400	100	0.4100	0.3600	0.3400	0.4500	MEALGTLPGHMTERFQEAIDNLAVLMRAETGSRPYKAAAAQLPETLETIMLLGLLGTVSLGIFFVLMRNKGIGKMGFGMVTLGASAWLMWLSEIEPARIA
2123	2182	NS4A	0.0057	0.0500	60	0.4500	0.3500	0.3667	0.4167	EALGTLPGHMTERFQEAIDNLAVLMRAETGSRPYKAAAAQLPETLETIMLLGLLGTVSLG
2123	2192	NS4A	0.0049	0.0429	70	0.4143	0.3571	0.3571	0.4429	EALGTLPGHMTERFQEAIDNLAVLMRAETGSRPYKAAAAQLPETLETIMLLGLLGTVSLGIFFVLMRNKG
2123	2202	NS4A	0.0052	0.0500	80	0.4250	0.3875	0.3625	0.4750	EALGTLPGHMTERFQEAIDNLAVLMRAETGSRPYKAAAAQLPETLETIMLLGLLGTVSLGIFFVLMRNKGIGKMGFGMVT
2123	2212	NS4A	0.0046	0.0444	90	0.4000	0.3667	0.3556	0.4667	EALGTLPGHMTERFQEAIDNLAVLMRAETGSRPYKAAAAQLPETLETIMLLGLLGTVSLGIFFVLMRNKGIGKMGFGMVTLGASAWLMWL
2123	2222	NS4A	0.0041	0.0400	100	0.4100	0.3600	0.3400	0.4500	EALGTLPGHMTERFQEAIDNLAVLMRAETGSRPYKAAAAQLPETLETIMLLGLLGTVSLGIFFVLMRNKGIGKMGFGMVTLGASAWLMWLSEIEPARIAC
2124	2183	NS4A	0.0024	0.0333	60	0.4500	0.3333	0.3667	0.4000	ALGTLPGHMTERFQEAIDNLAVLMRAETGSRPYKAAAAQLPETLETIMLLGLLGTVSLGI
2124	2193	NS4A	0.0020	0.0286	70	0.4286	0.3571	0.3714	0.4429	ALGTLPGHMTERFQEAIDNLAVLMRAETGSRPYKAAAAQLPETLETIMLLGLLGTVSLGIFFVLMRNKGI
2124	2203	NS4A	0.0027	0.0375	80	0.4250	0.3875	0.3625	0.4750	ALGTLPGHMTERFQEAIDNLAVLMRAETGSRPYKAAAAQLPETLETIMLLGLLGTVSLGIFFVLMRNKGIGKMGFGMVTL
2124	2213	NS4A	0.0024	0.0333	90	0.4000	0.3556	0.3556	0.4556	ALGTLPGHMTERFQEAIDNLAVLMRAETGSRPYKAAAAQLPETLETIMLLGLLGTVSLGIFFVLMRNKGIGKMGFGMVTLGASAWLMWLS
2124	2223	NS4A	0.0021	0.0300	100	0.4100	0.3500	0.3400	0.4400	ALGTLPGHMTERFQEAIDNLAVLMRAETGSRPYKAAAAQLPETLETIMLLGLLGTVSLGIFFVLMRNKGIGKMGFGMVTLGASAWLMWLSEIEPARIACV
2125	2184	NS4A	0.0024	0.0333	60	0.4500	0.3500	0.3667	0.4167	LGTLPGHMTERFQEAIDNLAVLMRAETGSRPYKAAAAQLPETLETIMLLGLLGTVSLGIF
2125	2194	NS4A	0.0020	0.0286	70	0.4429	0.3714	0.3714	0.4571	LGTLPGHMTERFQEAIDNLAVLMRAETGSRPYKAAAAQLPETLETIMLLGLLGTVSLGIFFVLMRNKGIG
2125	2204	NS4A	0.0027	0.0375	80	0.4250	0.3875	0.3625	0.4875	LGTLPGHMTERFQEAIDNLAVLMRAETGSRPYKAAAAQLPETLETIMLLGLLGTVSLGIFFVLMRNKGIGKMGFGMVTLG
2125	2214	NS4A	0.0024	0.0333	90	0.4111	0.3667	0.3556	0.4667	LGTLPGHMTERFQEAIDNLAVLMRAETGSRPYKAAAAQLPETLETIMLLGLLGTVSLGIFFVLMRNKGIGKMGFGMVTLGASAWLMWLSE
2125	2224	NS4A	0.0021	0.0300	100	0.4100	0.3600	0.3400	0.4500	LGTLPGHMTERFQEAIDNLAVLMRAETGSRPYKAAAAQLPETLETIMLLGLLGTVSLGIFFVLMRNKGIGKMGFGMVTLGASAWLMWLSEIEPARIACVL
2126	2185	NS4A	0.0024	0.0333	60	0.4500	0.3333	0.3500	0.4167	GTLPGHMTERFQEAIDNLAVLMRAETGSRPYKAAAAQLPETLETIMLLGLLGTVSLGIFF
2126	2195	NS4A	0.0020	0.0286	70	0.4571	0.3714	0.3571	0.4571	GTLPGHMTERFQEAIDNLAVLMRAETGSRPYKAAAAQLPETLETIMLLGLLGTVSLGIFFVLMRNKGIGK
2126	2205	NS4A	0.0027	0.0375	80	0.4250	0.3750	0.3500	0.4875	GTLPGHMTERFQEAIDNLAVLMRAETGSRPYKAAAAQLPETLETIMLLGLLGTVSLGIFFVLMRNKGIGKMGFGMVTLGA
2126	2215	NS4A	0.0024	0.0333	90	0.4222	0.3556	0.3444	0.4556	GTLPGHMTERFQEAIDNLAVLMRAETGSRPYKAAAAQLPETLETIMLLGLLGTVSLGIFFVLMRNKGIGKMGFGMVTLGASAWLMWLSEI
2126	2225	NS4A	0.0021	0.0300	100	0.4200	0.3500	0.3300	0.4400	GTLPGHMTERFQEAIDNLAVLMRAETGSRPYKAAAAQLPETLETIMLLGLLGTVSLGIFFVLMRNKGIGKMGFGMVTLGASAWLMWLSEIEPARIACVLI
2127	2186	NS4A	0.0024	0.0333	60	0.4333	0.3167	0.3500	0.4000	TLPGHMTERFQEAIDNLAVLMRAETGSRPYKAAAAQLPETLETIMLLGLLGTVSLGIFFV
2127	2196	NS4A	0.0020	0.0286	70	0.4571	0.3714	0.3714	0.4429	TLPGHMTERFQEAIDNLAVLMRAETGSRPYKAAAAQLPETLETIMLLGLLGTVSLGIFFVLMRNKGIGKM
2127	2206	NS4A	0.0027	0.0375	80	0.4250	0.3625	0.3500	0.4750	TLPGHMTERFQEAIDNLAVLMRAETGSRPYKAAAAQLPETLETIMLLGLLGTVSLGIFFVLMRNKGIGKMGFGMVTLGAS
2127	2216	NS4A	0.0024	0.0333	90	0.4222	0.3444	0.3444	0.4444	TLPGHMTERFQEAIDNLAVLMRAETGSRPYKAAAAQLPETLETIMLLGLLGTVSLGIFFVLMRNKGIGKMGFGMVTLGASAWLMWLSEIE
2127	2226	NS4A	0.0021	0.0300	100	0.4200	0.3500	0.3300	0.4300	TLPGHMTERFQEAIDNLAVLMRAETGSRPYKAAAAQLPETLETIMLLGLLGTVSLGIFFVLMRNKGIGKMGFGMVTLGASAWLMWLSEIEPARIACVLIV
2128	2187	NS4A	0.0024	0.0333	60	0.4333	0.3167	0.3500	0.4167	LPGHMTERFQEAIDNLAVLMRAETGSRPYKAAAAQLPETLETIMLLGLLGTVSLGIFFVL
2128	2197	NS4A	0.0020	0.0286	70	0.4571	0.3857	0.3714	0.4571	LPGHMTERFQEAIDNLAVLMRAETGSRPYKAAAAQLPETLETIMLLGLLGTVSLGIFFVLMRNKGIGKMG
2128	2207	NS4A	0.0027	0.0375	80	0.4250	0.3625	0.3500	0.4750	LPGHMTERFQEAIDNLAVLMRAETGSRPYKAAAAQLPETLETIMLLGLLGTVSLGIFFVLMRNKGIGKMGFGMVTLGASA
2128	2217	NS4A	0.0024	0.0333	90	0.4333	0.3444	0.3556	0.4444	LPGHMTERFQEAIDNLAVLMRAETGSRPYKAAAAQLPETLETIMLLGLLGTVSLGIFFVLMRNKGIGKMGFGMVTLGASAWLMWLSEIEP
2128	2227	NS4A	0.0021	0.0300	100	0.4200	0.3500	0.3300	0.4300	LPGHMTERFQEAIDNLAVLMRAETGSRPYKAAAAQLPETLETIMLLGLLGTVSLGIFFVLMRNKGIGKMGFGMVTLGASAWLMWLSEIEPARIACVLIVV
2129	2188	NS4A	0.0024	0.0333	60	0.4333	0.3333	0.3500	0.4333	PGHMTERFQEAIDNLAVLMRAETGSRPYKAAAAQLPETLETIMLLGLLGTVSLGIFFVLM
2129	2198	NS4A	0.0020	0.0286	70	0.4429	0.3857	0.3571	0.4571	PGHMTERFQEAIDNLAVLMRAETGSRPYKAAAAQLPETLETIMLLGLLGTVSLGIFFVLMRNKGIGKMGF
2129	2208	NS4A	0.0027	0.0375	80	0.4125	0.3625	0.3375	0.4750	PGHMTERFQEAIDNLAVLMRAETGSRPYKAAAAQLPETLETIMLLGLLGTVSLGIFFVLMRNKGIGKMGFGMVTLGASAW
2129	2218	NS4A	0.0024	0.0333	90	0.4222	0.3444	0.3444	0.4444	PGHMTERFQEAIDNLAVLMRAETGSRPYKAAAAQLPETLETIMLLGLLGTVSLGIFFVLMRNKGIGKMGFGMVTLGASAWLMWLSEIEPA
2129	2228	NS4A	0.0021	0.0300	100	0.4200	0.3500	0.3300	0.4300	PGHMTERFQEAIDNLAVLMRAETGSRPYKAAAAQLPETLETIMLLGLLGTVSLGIFFVLMRNKGIGKMGFGMVTLGASAWLMWLSEIEPARIACVLIVVF
2130	2179	NS4A	0.0029	0.0400	50	0.4600	0.3200	0.3600	0.3600	GHMTERFQEAIDNLAVLMRAETGSRPYKAAAAQLPETLETIMLLGLLGTV
2130	2189	NS4A	0.0024	0.0333	60	0.4167	0.3167	0.3333	0.4167	GHMTERFQEAIDNLAVLMRAETGSRPYKAAAAQLPETLETIMLLGLLGTVSLGIFFVLMR
2130	2199	NS4A	0.0031	0.0429	70	0.4429	0.3857	0.3429	0.4571	GHMTERFQEAIDNLAVLMRAETGSRPYKAAAAQLPETLETIMLLGLLGTVSLGIFFVLMRNKGIGKMGFG
2130	2209	NS4A	0.0027	0.0375	80	0.4125	0.3500	0.3375	0.4625	GHMTERFQEAIDNLAVLMRAETGSRPYKAAAAQLPETLETIMLLGLLGTVSLGIFFVLMRNKGIGKMGFGMVTLGASAWL
2130	2219	NS4A	0.0024	0.0333	90	0.4111	0.3333	0.3333	0.4333	GHMTERFQEAIDNLAVLMRAETGSRPYKAAAAQLPETLETIMLLGLLGTVSLGIFFVLMRNKGIGKMGFGMVTLGASAWLMWLSEIEPAR
2130	2229	NS4A	0.0021	0.0300	100	0.4100	0.3500	0.3200	0.4300	GHMTERFQEAIDNLAVLMRAETGSRPYKAAAAQLPETLETIMLLGLLGTVSLGIFFVLMRNKGIGKMGFGMVTLGASAWLMWLSEIEPARIACVLIVVFL
2131	2180	NS4A	0.0029	0.0400	50	0.4600	0.3200	0.3600	0.3800	HMTERFQEAIDNLAVLMRAETGSRPYKAAAAQLPETLETIMLLGLLGTVS
2131	2190	NS4A	0.0024	0.0333	60	0.4167	0.3167	0.3333	0.4167	HMTERFQEAIDNLAVLMRAETGSRPYKAAAAQLPETLETIMLLGLLGTVSLGIFFVLMRN
2131	2200	NS4A	0.0031	0.0429	70	0.4429	0.3857	0.3571	0.4571	HMTERFQEAIDNLAVLMRAETGSRPYKAAAAQLPETLETIMLLGLLGTVSLGIFFVLMRNKGIGKMGFGM
2131	2210	NS4A	0.0027	0.0375	80	0.4125	0.3500	0.3500	0.4625	HMTERFQEAIDNLAVLMRAETGSRPYKAAAAQLPETLETIMLLGLLGTVSLGIFFVLMRNKGIGKMGFGMVTLGASAWLM
2131	2220	NS4A	0.0024	0.0333	90	0.4222	0.3444	0.3444	0.4444	HMTERFQEAIDNLAVLMRAETGSRPYKAAAAQLPETLETIMLLGLLGTVSLGIFFVLMRNKGIGKMGFGMVTLGASAWLMWLSEIEPARI
2131	2230	NS4A	0.0021	0.0300	100	0.4200	0.3600	0.3300	0.4400	HMTERFQEAIDNLAVLMRAETGSRPYKAAAAQLPETLETIMLLGLLGTVSLGIFFVLMRNKGIGKMGFGMVTLGASAWLMWLSEIEPARIACVLIVVFLL
2132	2181	NS4A	0.0029	0.0400	50	0.4400	0.3000	0.3600	0.3800	MTERFQEAIDNLAVLMRAETGSRPYKAAAAQLPETLETIMLLGLLGTVSL
2132	2191	NS4A	0.0024	0.0333	60	0.4333	0.3167	0.3500	0.4167	MTERFQEAIDNLAVLMRAETGSRPYKAAAAQLPETLETIMLLGLLGTVSLGIFFVLMRNK
2132	2201	NS4A	0.0031	0.0429	70	0.4429	0.3714	0.3571	0.4571	MTERFQEAIDNLAVLMRAETGSRPYKAAAAQLPETLETIMLLGLLGTVSLGIFFVLMRNKGIGKMGFGMV
2132	2211	NS4A	0.0027	0.0375	80	0.4250	0.3500	0.3500	0.4625	MTERFQEAIDNLAVLMRAETGSRPYKAAAAQLPETLETIMLLGLLGTVSLGIFFVLMRNKGIGKMGFGMVTLGASAWLMW
2132	2221	NS4A	0.0024	0.0333	90	0.4333	0.3444	0.3444	0.4444	MTERFQEAIDNLAVLMRAETGSRPYKAAAAQLPETLETIMLLGLLGTVSLGIFFVLMRNKGIGKMGFGMVTLGASAWLMWLSEIEPARIA
2132	2231	NS4A	0.0021	0.0300	100	0.4200	0.3500	0.3300	0.4300	MTERFQEAIDNLAVLMRAETGSRPYKAAAAQLPETLETIMLLGLLGTVSLGIFFVLMRNKGIGKMGFGMVTLGASAWLMWLSEIEPARIACVLIVVFLLL
2133	2192	NS4A	0.0024	0.0333	60	0.4500	0.3333	0.3667	0.4333	TERFQEAIDNLAVLMRAETGSRPYKAAAAQLPETLETIMLLGLLGTVSLGIFFVLMRNKG
2133	2202	NS4A	0.0031	0.0429	70	0.4571	0.3714	0.3714	0.4714	TERFQEAIDNLAVLMRAETGSRPYKAAAAQLPETLETIMLLGLLGTVSLGIFFVLMRNKGIGKMGFGMVT
2133	2212	NS4A	0.0027	0.0375	80	0.4250	0.3500	0.3625	0.4625	TERFQEAIDNLAVLMRAETGSRPYKAAAAQLPETLETIMLLGLLGTVSLGIFFVLMRNKGIGKMGFGMVTLGASAWLMWL
2133	2222	NS4A	0.0024	0.0333	90	0.4333	0.3444	0.3444	0.4444	TERFQEAIDNLAVLMRAETGSRPYKAAAAQLPETLETIMLLGLLGTVSLGIFFVLMRNKGIGKMGFGMVTLGASAWLMWLSEIEPARIAC
2133	2232	NS4A	0.0021	0.0300	100	0.4300	0.3600	0.3400	0.4300	TERFQEAIDNLAVLMRAETGSRPYKAAAAQLPETLETIMLLGLLGTVSLGIFFVLMRNKGIGKMGFGMVTLGASAWLMWLSEIEPARIACVLIVVFLLLV
2134	2203	NS4A	0.0031	0.0429	70	0.4571	0.3857	0.3714	0.4857	ERFQEAIDNLAVLMRAETGSRPYKAAAAQLPETLETIMLLGLLGTVSLGIFFVLMRNKGIGKMGFGMVTL
2134	2213	NS4A	0.0027	0.0375	80	0.4250	0.3500	0.3625	0.4625	ERFQEAIDNLAVLMRAETGSRPYKAAAAQLPETLETIMLLGLLGTVSLGIFFVLMRNKGIGKMGFGMVTLGASAWLMWLS
2134	2223	NS4A	0.0024	0.0333	90	0.4333	0.3444	0.3444	0.4444	ERFQEAIDNLAVLMRAETGSRPYKAAAAQLPETLETIMLLGLLGTVSLGIFFVLMRNKGIGKMGFGMVTLGASAWLMWLSEIEPARIACV
2134	2233	NS4A	0.0021	0.0300	100	0.4300	0.3700	0.3500	0.4400	ERFQEAIDNLAVLMRAETGSRPYKAAAAQLPETLETIMLLGLLGTVSLGIFFVLMRNKGIGKMGFGMVTLGASAWLMWLSEIEPARIACVLIVVFLLLVV
2135	2204	NS4A	0.0031	0.0429	70	0.4571	0.3714	0.3714	0.5000	RFQEAIDNLAVLMRAETGSRPYKAAAAQLPETLETIMLLGLLGTVSLGIFFVLMRNKGIGKMGFGMVTLG
2135	2214	NS4A	0.0027	0.0375	80	0.4375	0.3500	0.3625	0.4750	RFQEAIDNLAVLMRAETGSRPYKAAAAQLPETLETIMLLGLLGTVSLGIFFVLMRNKGIGKMGFGMVTLGASAWLMWLSE
2135	2224	NS4A	0.0024	0.0333	90	0.4333	0.3444	0.3444	0.4556	RFQEAIDNLAVLMRAETGSRPYKAAAAQLPETLETIMLLGLLGTVSLGIFFVLMRNKGIGKMGFGMVTLGASAWLMWLSEIEPARIACVL
2135	2234	NS4A	0.0021	0.0300	100	0.4400	0.3700	0.3500	0.4500	RFQEAIDNLAVLMRAETGSRPYKAAAAQLPETLETIMLLGLLGTVSLGIFFVLMRNKGIGKMGFGMVTLGASAWLMWLSEIEPARIACVLIVVFLLLVVL
2136	2215	NS4A	0.0027	0.0375	80	0.4375	0.3500	0.3625	0.4750	FQEAIDNLAVLMRAETGSRPYKAAAAQLPETLETIMLLGLLGTVSLGIFFVLMRNKGIGKMGFGMVTLGASAWLMWLSEI
2136	2225	NS4A	0.0024	0.0333	90	0.4333	0.3444	0.3444	0.4556	FQEAIDNLAVLMRAETGSRPYKAAAAQLPETLETIMLLGLLGTVSLGIFFVLMRNKGIGKMGFGMVTLGASAWLMWLSEIEPARIACVLI
2136	2235	NS4A	0.0021	0.0300	100	0.4400	0.3800	0.3500	0.4600	FQEAIDNLAVLMRAETGSRPYKAAAAQLPETLETIMLLGLLGTVSLGIFFVLMRNKGIGKMGFGMVTLGASAWLMWLSEIEPARIACVLIVVFLLLVVLI
2137	2216	NS4A	0.0027	0.0375	80	0.4375	0.3500	0.3625	0.4750	QEAIDNLAVLMRAETGSRPYKAAAAQLPETLETIMLLGLLGTVSLGIFFVLMRNKGIGKMGFGMVTLGASAWLMWLSEIE
2137	2226	NS4A	0.0024	0.0333	90	0.4333	0.3556	0.3444	0.4556	QEAIDNLAVLMRAETGSRPYKAAAAQLPETLETIMLLGLLGTVSLGIFFVLMRNKGIGKMGFGMVTLGASAWLMWLSEIEPARIACVLIV
2137	2236	NS4A	0.0021	0.0300	100	0.4500	0.3900	0.3600	0.4700	QEAIDNLAVLMRAETGSRPYKAAAAQLPETLETIMLLGLLGTVSLGIFFVLMRNKGIGKMGFGMVTLGASAWLMWLSEIEPARIACVLIVVFLLLVVLIP
2138	2217	NS4A	0.0027	0.0375	80	0.4500	0.3500	0.3750	0.4750	EAIDNLAVLMRAETGSRPYKAAAAQLPETLETIMLLGLLGTVSLGIFFVLMRNKGIGKMGFGMVTLGASAWLMWLSEIEP
2138	2227	NS4A	0.0024	0.0333	90	0.4333	0.3556	0.3444	0.4556	EAIDNLAVLMRAETGSRPYKAAAAQLPETLETIMLLGLLGTVSLGIFFVLMRNKGIGKMGFGMVTLGASAWLMWLSEIEPARIACVLIVV
2138	2237	NS4A	0.0021	0.0300	100	0.4600	0.4000	0.3700	0.4800	EAIDNLAVLMRAETGSRPYKAAAAQLPETLETIMLLGLLGTVSLGIFFVLMRNKGIGKMGFGMVTLGASAWLMWLSEIEPARIACVLIVVFLLLVVLIPE
2139	2208	NS4A	0.0031	0.0429	70	0.4429	0.3571	0.3571	0.5000	AIDNLAVLMRAETGSRPYKAAAAQLPETLETIMLLGLLGTVSLGIFFVLMRNKGIGKMGFGMVTLGASAW
2139	2218	NS4A	0.0027	0.0375	80	0.4500	0.3375	0.3625	0.4625	AIDNLAVLMRAETGSRPYKAAAAQLPETLETIMLLGLLGTVSLGIFFVLMRNKGIGKMGFGMVTLGASAWLMWLSEIEPA
2139	2228	NS4A	0.0024	0.0333	90	0.4444	0.3444	0.3444	0.4444	AIDNLAVLMRAETGSRPYKAAAAQLPETLETIMLLGLLGTVSLGIFFVLMRNKGIGKMGFGMVTLGASAWLMWLSEIEPARIACVLIVVF
2139	2238	NS4A	0.0021	0.0300	100	0.4700	0.4000	0.3700	0.4800	AIDNLAVLMRAETGSRPYKAAAAQLPETLETIMLLGLLGTVSLGIFFVLMRNKGIGKMGFGMVTLGASAWLMWLSEIEPARIACVLIVVFLLLVVLIPEP
2140	2189	NS4A	0.0029	0.0400	50	0.4600	0.3000	0.3600	0.4400	IDNLAVLMRAETGSRPYKAAAAQLPETLETIMLLGLLGTVSLGIFFVLMR
2140	2199	NS4A	0.0036	0.0500	60	0.4833	0.3833	0.3667	0.4833	IDNLAVLMRAETGSRPYKAAAAQLPETLETIMLLGLLGTVSLGIFFVLMRNKGIGKMGFG
2140	2209	NS4A	0.0031	0.0429	70	0.4429	0.3429	0.3571	0.4857	IDNLAVLMRAETGSRPYKAAAAQLPETLETIMLLGLLGTVSLGIFFVLMRNKGIGKMGFGMVTLGASAWL
2140	2219	NS4A	0.0027	0.0375	80	0.4375	0.3250	0.3500	0.4500	IDNLAVLMRAETGSRPYKAAAAQLPETLETIMLLGLLGTVSLGIFFVLMRNKGIGKMGFGMVTLGASAWLMWLSEIEPAR
2140	2229	NS4A	0.0024	0.0333	90	0.4333	0.3444	0.3333	0.4444	IDNLAVLMRAETGSRPYKAAAAQLPETLETIMLLGLLGTVSLGIFFVLMRNKGIGKMGFGMVTLGASAWLMWLSEIEPARIACVLIVVFL
2140	2239	NS4A	0.0021	0.0300	100	0.4700	0.4000	0.3600	0.4800	IDNLAVLMRAETGSRPYKAAAAQLPETLETIMLLGLLGTVSLGIFFVLMRNKGIGKMGFGMVTLGASAWLMWLSEIEPARIACVLIVVFLLLVVLIPEPE
2141	2190	NS4A	0.0029	0.0400	50	0.4600	0.3000	0.3600	0.4400	DNLAVLMRAETGSRPYKAAAAQLPETLETIMLLGLLGTVSLGIFFVLMRN
2141	2210	NS4A	0.0031	0.0429	70	0.4429	0.3429	0.3714	0.4857	DNLAVLMRAETGSRPYKAAAAQLPETLETIMLLGLLGTVSLGIFFVLMRNKGIGKMGFGMVTLGASAWLM
2141	2220	NS4A	0.0027	0.0375	80	0.4500	0.3375	0.3625	0.4625	DNLAVLMRAETGSRPYKAAAAQLPETLETIMLLGLLGTVSLGIFFVLMRNKGIGKMGFGMVTLGASAWLMWLSEIEPARI
2141	2230	NS4A	0.0024	0.0333	90	0.4444	0.3556	0.3444	0.4556	DNLAVLMRAETGSRPYKAAAAQLPETLETIMLLGLLGTVSLGIFFVLMRNKGIGKMGFGMVTLGASAWLMWLSEIEPARIACVLIVVFLL
2141	2240	NS4A	0.0021	0.0300	100	0.4800	0.4100	0.3600	0.4900	DNLAVLMRAETGSRPYKAAAAQLPETLETIMLLGLLGTVSLGIFFVLMRNKGIGKMGFGMVTLGASAWLMWLSEIEPARIACVLIVVFLLLVVLIPEPEK
2142	2191	NS4A	0.0029	0.0400	50	0.4600	0.3000	0.3600	0.4400	NLAVLMRAETGSRPYKAAAAQLPETLETIMLLGLLGTVSLGIFFVLMRNK
2142	2201	NS4A	0.0036	0.0500	60	0.4667	0.3667	0.3667	0.4833	NLAVLMRAETGSRPYKAAAAQLPETLETIMLLGLLGTVSLGIFFVLMRNKGIGKMGFGMV
2142	2211	NS4A	0.0031	0.0429	70	0.4429	0.3429	0.3571	0.4857	NLAVLMRAETGSRPYKAAAAQLPETLETIMLLGLLGTVSLGIFFVLMRNKGIGKMGFGMVTLGASAWLMW
2142	2221	NS4A	0.0027	0.0375	80	0.4500	0.3375	0.3500	0.4625	NLAVLMRAETGSRPYKAAAAQLPETLETIMLLGLLGTVSLGIFFVLMRNKGIGKMGFGMVTLGASAWLMWLSEIEPARIA
2142	2231	NS4A	0.0024	0.0333	90	0.4333	0.3444	0.3333	0.4444	NLAVLMRAETGSRPYKAAAAQLPETLETIMLLGLLGTVSLGIFFVLMRNKGIGKMGFGMVTLGASAWLMWLSEIEPARIACVLIVVFLLL
2142	2241	NS4A	0.0021	0.0300	100	0.4800	0.4100	0.3600	0.4900	NLAVLMRAETGSRPYKAAAAQLPETLETIMLLGLLGTVSLGIFFVLMRNKGIGKMGFGMVTLGASAWLMWLSEIEPARIACVLIVVFLLLVVLIPEPEKQ
2143	2212	NS4A	0.0031	0.0429	70	0.4286	0.3429	0.3714	0.4857	LAVLMRAETGSRPYKAAAAQLPETLETIMLLGLLGTVSLGIFFVLMRNKGIGKMGFGMVTLGASAWLMWL
2143	2222	NS4A	0.0027	0.0375	80	0.4375	0.3375	0.3500	0.4625	LAVLMRAETGSRPYKAAAAQLPETLETIMLLGLLGTVSLGIFFVLMRNKGIGKMGFGMVTLGASAWLMWLSEIEPARIAC
2143	2232	NS4A	0.0024	0.0333	90	0.4333	0.3556	0.3444	0.4444	LAVLMRAETGSRPYKAAAAQLPETLETIMLLGLLGTVSLGIFFVLMRNKGIGKMGFGMVTLGASAWLMWLSEIEPARIACVLIVVFLLLV
2143	2242	NS4A	0.0021	0.0300	100	0.4800	0.4200	0.3700	0.5000	LAVLMRAETGSRPYKAAAAQLPETLETIMLLGLLGTVSLGIFFVLMRNKGIGKMGFGMVTLGASAWLMWLSEIEPARIACVLIVVFLLLVVLIPEPEKQR
2144	2213	NS4A	0.0031	0.0429	70	0.4286	0.3429	0.3714	0.4857	AVLMRAETGSRPYKAAAAQLPETLETIMLLGLLGTVSLGIFFVLMRNKGIGKMGFGMVTLGASAWLMWLS
2144	2223	NS4A	0.0027	0.0375	80	0.4375	0.3375	0.3500	0.4625	AVLMRAETGSRPYKAAAAQLPETLETIMLLGLLGTVSLGIFFVLMRNKGIGKMGFGMVTLGASAWLMWLSEIEPARIACV
2144	2233	NS4A	0.0024	0.0333	90	0.4333	0.3667	0.3556	0.4556	AVLMRAETGSRPYKAAAAQLPETLETIMLLGLLGTVSLGIFFVLMRNKGIGKMGFGMVTLGASAWLMWLSEIEPARIACVLIVVFLLLVV
2145	2214	NS4A	0.0031	0.0429	70	0.4429	0.3571	0.3714	0.5000	VLMRAETGSRPYKAAAAQLPETLETIMLLGLLGTVSLGIFFVLMRNKGIGKMGFGMVTLGASAWLMWLSE
2145	2224	NS4A	0.0027	0.0375	80	0.4375	0.3500	0.3500	0.4750	VLMRAETGSRPYKAAAAQLPETLETIMLLGLLGTVSLGIFFVLMRNKGIGKMGFGMVTLGASAWLMWLSEIEPARIACVL
2145	2234	NS4A	0.0024	0.0333	90	0.4444	0.3778	0.3556	0.4667	VLMRAETGSRPYKAAAAQLPETLETIMLLGLLGTVSLGIFFVLMRNKGIGKMGFGMVTLGASAWLMWLSEIEPARIACVLIVVFLLLVVL
2146	2215	NS4A	0.0031	0.0429	70	0.4571	0.3571	0.3571	0.4857	LMRAETGSRPYKAAAAQLPETLETIMLLGLLGTVSLGIFFVLMRNKGIGKMGFGMVTLGASAWLMWLSEI
2146	2225	NS4A	0.0027	0.0375	80	0.4500	0.3500	0.3375	0.4625	LMRAETGSRPYKAAAAQLPETLETIMLLGLLGTVSLGIFFVLMRNKGIGKMGFGMVTLGASAWLMWLSEIEPARIACVLI
2146	2235	NS4A	0.0024	0.0333	90	0.4556	0.3889	0.3444	0.4667	LMRAETGSRPYKAAAAQLPETLETIMLLGLLGTVSLGIFFVLMRNKGIGKMGFGMVTLGASAWLMWLSEIEPARIACVLIVVFLLLVVLI
2147	2216	NS4A	0.0031	0.0429	70	0.4429	0.3571	0.3429	0.4857	MRAETGSRPYKAAAAQLPETLETIMLLGLLGTVSLGIFFVLMRNKGIGKMGFGMVTLGASAWLMWLSEIE
2147	2226	NS4A	0.0027	0.0375	80	0.4375	0.3625	0.3250	0.4625	MRAETGSRPYKAAAAQLPETLETIMLLGLLGTVSLGIFFVLMRNKGIGKMGFGMVTLGASAWLMWLSEIEPARIACVLIV
2147	2236	NS4A	0.0024	0.0333	90	0.4556	0.4000	0.3556	0.4778	MRAETGSRPYKAAAAQLPETLETIMLLGLLGTVSLGIFFVLMRNKGIGKMGFGMVTLGASAWLMWLSEIEPARIACVLIVVFLLLVVLIP
2148	2217	NS4A	0.0031	0.0429	70	0.4571	0.3571	0.3571	0.4857	RAETGSRPYKAAAAQLPETLETIMLLGLLGTVSLGIFFVLMRNKGIGKMGFGMVTLGASAWLMWLSEIEP
2148	2227	NS4A	0.0027	0.0375	80	0.4375	0.3625	0.3250	0.4625	RAETGSRPYKAAAAQLPETLETIMLLGLLGTVSLGIFFVLMRNKGIGKMGFGMVTLGASAWLMWLSEIEPARIACVLIVV
2148	2237	NS4A	0.0024	0.0333	90	0.4667	0.4111	0.3667	0.4889	RAETGSRPYKAAAAQLPETLETIMLLGLLGTVSLGIFFVLMRNKGIGKMGFGMVTLGASAWLMWLSEIEPARIACVLIVVFLLLVVLIPE
2149	2218	NS4A	0.0031	0.0429	70	0.4571	0.3571	0.3571	0.4857	AETGSRPYKAAAAQLPETLETIMLLGLLGTVSLGIFFVLMRNKGIGKMGFGMVTLGASAWLMWLSEIEPA
2149	2228	NS4A	0.0027	0.0375	80	0.4500	0.3625	0.3375	0.4625	AETGSRPYKAAAAQLPETLETIMLLGLLGTVSLGIFFVLMRNKGIGKMGFGMVTLGASAWLMWLSEIEPARIACVLIVVF
2149	2238	NS4A	0.0024	0.0333	90	0.4778	0.4222	0.3778	0.5000	AETGSRPYKAAAAQLPETLETIMLLGLLGTVSLGIFFVLMRNKGIGKMGFGMVTLGASAWLMWLSEIEPARIACVLIVVFLLLVVLIPEP
2150	2219	NS4A	0.0031	0.0429	70	0.4571	0.3429	0.3571	0.4714	ETGSRPYKAAAAQLPETLETIMLLGLLGTVSLGIFFVLMRNKGIGKMGFGMVTLGASAWLMWLSEIEPAR
2150	2229	NS4A	0.0027	0.0375	80	0.4500	0.3625	0.3375	0.4625	ETGSRPYKAAAAQLPETLETIMLLGLLGTVSLGIFFVLMRNKGIGKMGFGMVTLGASAWLMWLSEIEPARIACVLIVVFL
2150	2239	NS4A	0.0024	0.0333	90	0.4889	0.4222	0.3778	0.5000	ETGSRPYKAAAAQLPETLETIMLLGLLGTVSLGIFFVLMRNKGIGKMGFGMVTLGASAWLMWLSEIEPARIACVLIVVFLLLVVLIPEPE
2151	2220	NS4A	0.0031	0.0429	70	0.4571	0.3429	0.3571	0.4714	TGSRPYKAAAAQLPETLETIMLLGLLGTVSLGIFFVLMRNKGIGKMGFGMVTLGASAWLMWLSEIEPARI
2151	2230	NS4A	0.0027	0.0375	80	0.4500	0.3625	0.3375	0.4625	TGSRPYKAAAAQLPETLETIMLLGLLGTVSLGIFFVLMRNKGIGKMGFGMVTLGASAWLMWLSEIEPARIACVLIVVFLL
2151	2240	NS4A	0.0024	0.0333	90	0.4889	0.4222	0.3667	0.5000	TGSRPYKAAAAQLPETLETIMLLGLLGTVSLGIFFVLMRNKGIGKMGFGMVTLGASAWLMWLSEIEPARIACVLIVVFLLLVVLIPEPEK
2152	2221	NS4A	0.0031	0.0429	70	0.4714	0.3571	0.3571	0.4857	GSRPYKAAAAQLPETLETIMLLGLLGTVSLGIFFVLMRNKGIGKMGFGMVTLGASAWLMWLSEIEPARIA
2152	2231	NS4A	0.0027	0.0375	80	0.4500	0.3625	0.3375	0.4625	GSRPYKAAAAQLPETLETIMLLGLLGTVSLGIFFVLMRNKGIGKMGFGMVTLGASAWLMWLSEIEPARIACVLIVVFLLL
2153	2222	NS4A	0.0031	0.0429	70	0.4571	0.3429	0.3429	0.4714	SRPYKAAAAQLPETLETIMLLGLLGTVSLGIFFVLMRNKGIGKMGFGMVTLGASAWLMWLSEIEPARIAC
2153	2232	NS4A	0.0027	0.0375	80	0.4500	0.3625	0.3375	0.4500	SRPYKAAAAQLPETLETIMLLGLLGTVSLGIFFVLMRNKGIGKMGFGMVTLGASAWLMWLSEIEPARIACVLIVVFLLLV
2154	2223	NS4A	0.0031	0.0429	70	0.4571	0.3429	0.3286	0.4714	RPYKAAAAQLPETLETIMLLGLLGTVSLGIFFVLMRNKGIGKMGFGMVTLGASAWLMWLSEIEPARIACV
2154	2233	NS4A	0.0027	0.0375	80	0.4500	0.3750	0.3375	0.4625	RPYKAAAAQLPETLETIMLLGLLGTVSLGIFFVLMRNKGIGKMGFGMVTLGASAWLMWLSEIEPARIACVLIVVFLLLVV
2155	2224	NS4A	0.0031	0.0429	70	0.4429	0.3571	0.3143	0.4714	PYKAAAAQLPETLETIMLLGLLGTVSLGIFFVLMRNKGIGKMGFGMVTLGASAWLMWLSEIEPARIACVL
2155	2234	NS4A	0.0027	0.0375	80	0.4500	0.3875	0.3250	0.4625	PYKAAAAQLPETLETIMLLGLLGTVSLGIFFVLMRNKGIGKMGFGMVTLGASAWLMWLSEIEPARIACVLIVVFLLLVVL
2156	2225	NS4A	0.0031	0.0429	70	0.4571	0.3571	0.3143	0.4714	YKAAAAQLPETLETIMLLGLLGTVSLGIFFVLMRNKGIGKMGFGMVTLGASAWLMWLSEIEPARIACVLI
2156	2235	NS4A	0.0027	0.0375	80	0.4625	0.4000	0.3375	0.4750	YKAAAAQLPETLETIMLLGLLGTVSLGIFFVLMRNKGIGKMGFGMVTLGASAWLMWLSEIEPARIACVLIVVFLLLVVLI
2157	2226	NS4A	0.0031	0.0429	70	0.4429	0.3714	0.3000	0.4714	KAAAAQLPETLETIMLLGLLGTVSLGIFFVLMRNKGIGKMGFGMVTLGASAWLMWLSEIEPARIACVLIV
2157	2236	NS4A	0.0027	0.0375	80	0.4625	0.4125	0.3375	0.4875	KAAAAQLPETLETIMLLGLLGTVSLGIFFVLMRNKGIGKMGFGMVTLGASAWLMWLSEIEPARIACVLIVVFLLLVVLIP
2158	2227	NS4A	0.0020	0.0286	70	0.4429	0.3714	0.3000	0.4714	AAAAQLPETLETIMLLGLLGTVSLGIFFVLMRNKGIGKMGFGMVTLGASAWLMWLSEIEPARIACVLIVV
2159	2228	NS4A	0.0020	0.0286	70	0.4571	0.3714	0.3143	0.4714	AAAQLPETLETIMLLGLLGTVSLGIFFVLMRNKGIGKMGFGMVTLGASAWLMWLSEIEPARIACVLIVVF
2160	2219	NS4A	0.0024	0.0333	60	0.4500	0.3500	0.3167	0.4833	AAQLPETLETIMLLGLLGTVSLGIFFVLMRNKGIGKMGFGMVTLGASAWLMWLSEIEPAR
2160	2229	NS4A	0.0020	0.0286	70	0.4429	0.3714	0.3000	0.4714	AAQLPETLETIMLLGLLGTVSLGIFFVLMRNKGIGKMGFGMVTLGASAWLMWLSEIEPARIACVLIVVFL
2161	2230	NS4A	0.0020	0.0286	70	0.4571	0.3857	0.3143	0.4857	AQLPETLETIMLLGLLGTVSLGIFFVLMRNKGIGKMGFGMVTLGASAWLMWLSEIEPARIACVLIVVFLL
2162	2231	NS4A	0.0020	0.0286	70	0.4571	0.3857	0.3143	0.4857	QLPETLETIMLLGLLGTVSLGIFFVLMRNKGIGKMGFGMVTLGASAWLMWLSEIEPARIACVLIVVFLLL
2163	2232	NS4A	0.0010	0.0143	70	0.4714	0.4000	0.3286	0.4857	LPETLETIMLLGLLGTVSLGIFFVLMRNKGIGKMGFGMVTLGASAWLMWLSEIEPARIACVLIVVFLLLV
2164	2233	NS4A	0.0010	0.0143	70	0.4571	0.4000	0.3429	0.4857	PETLETIMLLGLLGTVSLGIFFVLMRNKGIGKMGFGMVTLGASAWLMWLSEIEPARIACVLIVVFLLLVV
2165	2234	NS4A	0.0010	0.0143	70	0.4571	0.4000	0.3286	0.4857	ETLETIMLLGLLGTVSLGIFFVLMRNKGIGKMGFGMVTLGASAWLMWLSEIEPARIACVLIVVFLLLVVL
2166	2235	NS4A	0.0010	0.0143	70	0.4571	0.4143	0.3286	0.5000	TLETIMLLGLLGTVSLGIFFVLMRNKGIGKMGFGMVTLGASAWLMWLSEIEPARIACVLIVVFLLLVVLI
2168	2227	NS4A	0.0012	0.0167	60	0.4333	0.3667	0.3000	0.4833	ETIMLLGLLGTVSLGIFFVLMRNKGIGKMGFGMVTLGASAWLMWLSEIEPARIACVLIVV
2169	2228	NS4A	0.0012	0.0167	60	0.4333	0.3500	0.3167	0.4833	TIMLLGLLGTVSLGIFFVLMRNKGIGKMGFGMVTLGASAWLMWLSEIEPARIACVLIVVF
2170	2229	NS4A	0.0012	0.0167	60	0.4167	0.3500	0.3167	0.4833	IMLLGLLGTVSLGIFFVLMRNKGIGKMGFGMVTLGASAWLMWLSEIEPARIACVLIVVFL
2171	2230	NS4A	0.0012	0.0167	60	0.4333	0.3500	0.3333	0.4833	MLLGLLGTVSLGIFFVLMRNKGIGKMGFGMVTLGASAWLMWLSEIEPARIACVLIVVFLL
2172	2231	NS4A	0.0012	0.0167	60	0.4167	0.3500	0.3167	0.4833	LLGLLGTVSLGIFFVLMRNKGIGKMGFGMVTLGASAWLMWLSEIEPARIACVLIVVFLLL
2173	2232	NS4A	0.0012	0.0167	60	0.4167	0.3500	0.3167	0.4667	LGLLGTVSLGIFFVLMRNKGIGKMGFGMVTLGASAWLMWLSEIEPARIACVLIVVFLLLV
2174	2233	NS4A	0.0012	0.0167	60	0.4167	0.3667	0.3333	0.4833	GLLGTVSLGIFFVLMRNKGIGKMGFGMVTLGASAWLMWLSEIEPARIACVLIVVFLLLVV
2177	2226	NS4A	0.0014	0.0200	50	0.4000	0.3600	0.3000	0.4800	GTVSLGIFFVLMRNKGIGKMGFGMVTLGASAWLMWLSEIEPARIACVLIV
2178	2227	NS4A	0.0014	0.0200	50	0.3800	0.3600	0.2800	0.4800	TVSLGIFFVLMRNKGIGKMGFGMVTLGASAWLMWLSEIEPARIACVLIVV
2179	2228	NS4A	0.0014	0.0200	50	0.4000	0.3600	0.3000	0.4800	VSLGIFFVLMRNKGIGKMGFGMVTLGASAWLMWLSEIEPARIACVLIVVF
2182	2231	NS4A	0.0014	0.0200	50	0.4200	0.4000	0.3200	0.4800	GIFFVLMRNKGIGKMGFGMVTLGASAWLMWLSEIEPARIACVLIVVFLLL
2183	2232	NS4A	0.0014	0.0200	50	0.4200	0.4000	0.3200	0.4600	IFFVLMRNKGIGKMGFGMVTLGASAWLMWLSEIEPARIACVLIVVFLLLV
2184	2233	NS4A	0.0014	0.0200	50	0.4000	0.4200	0.3400	0.4800	FFVLMRNKGIGKMGFGMVTLGASAWLMWLSEIEPARIACVLIVVFLLLVV
2185	2234	NS4A	0.0014	0.0200	50	0.4000	0.4200	0.3400	0.4800	FVLMRNKGIGKMGFGMVTLGASAWLMWLSEIEPARIACVLIVVFLLLVVL
2186	2235	NS4A	0.0014	0.0200	50	0.4200	0.4400	0.3600	0.4800	VLMRNKGIGKMGFGMVTLGASAWLMWLSEIEPARIACVLIVVFLLLVVLI
2188	2227	NS4A	0.0018	0.0250	40	0.4000	0.4000	0.3250	0.4500	MRNKGIGKMGFGMVTLGASAWLMWLSEIEPARIACVLIVV
2189	2228	NS4A	0.0018	0.0250	40	0.4000	0.3750	0.3250	0.4250	RNKGIGKMGFGMVTLGASAWLMWLSEIEPARIACVLIVVF
2190	2229	NS4A	0.0018	0.0250	40	0.4000	0.4000	0.3250	0.4500	NKGIGKMGFGMVTLGASAWLMWLSEIEPARIACVLIVVFL
2192	2231	NS4A	0.0018	0.0250	40	0.4000	0.4000	0.3250	0.4500	GIGKMGFGMVTLGASAWLMWLSEIEPARIACVLIVVFLLL
2193	2232	NS4A	0.0018	0.0250	40	0.4000	0.4000	0.3250	0.4250	IGKMGFGMVTLGASAWLMWLSEIEPARIACVLIVVFLLLV
2203	2222	NS4A	0.0000	0.0000	20	0.4000	0.2500	0.3000	0.3500	LGASAWLMWLSEIEPARIAC
2207	2226	NS4A	0.0000	0.0000	20	0.4000	0.3000	0.3000	0.2500	AWLMWLSEIEPARIACVLIV
2208	2227	NS4A	0.0000	0.0000	20	0.4000	0.3000	0.3000	0.2500	WLMWLSEIEPARIACVLIVV
2210	2229	NS4A	0.0000	0.0000	20	0.4000	0.3500	0.3000	0.3000	MWLSEIEPARIACVLIVVFL
2212	2231	NS4A	0.0000	0.0000	20	0.4000	0.3500	0.3000	0.3000	LSEIEPARIACVLIVVFLLL
2316	2335	NS4B	0.0000	0.0000	20	0.4000	0.3500	0.3000	0.4000	TPAVQHAVTTSYNNYSLMAM
2317	2326	NS4B	0.0000	0.0000	10	0.3000	0.3000	0.2000	0.3000	PAVQHAVTTS
2318	2323	NS4B	0.0000	0.0000	6	0.1667	0.1667	0.1667	0.1667	AVQHAV
2318	2327	NS4B	0.0000	0.0000	10	0.2000	0.3000	0.2000	0.3000	AVQHAVTTSY
2318	2337	NS4B	0.0000	0.0000	20	0.3500	0.2500	0.3000	0.3000	AVQHAVTTSYNNYSLMAMAT
2319	2328	NS4B	0.0000	0.0000	10	0.2000	0.3000	0.2000	0.3000	VQHAVTTSYN
2319	2338	NS4B	0.0000	0.0000	20	0.4000	0.3000	0.3000	0.3500	VQHAVTTSYNNYSLMAMATQ
2418	2427	NS4B	0.0000	0.0000	10	0.3000	0.3000	0.2000	0.3000	VVTDIDTMTI
2419	2428	NS4B	0.0000	0.0000	10	0.3000	0.2000	0.2000	0.2000	VTDIDTMTID
2422	2427	NS4B	0.0000	0.0000	6	0.1667	0.0000	0.1667	0.0000	IDTMTI
2423	2428	NS4B	0.0000	0.0000	6	0.3333	0.0000	0.0000	0.0000	DTMTID
2453	2458	NS4B	0.0000	0.0000	6	0.3333	0.0000	0.1667	0.0000	TAWGWG
2453	2462	NS4B	0.0000	0.0000	10	0.4000	0.3000	0.2000	0.3000	TAWGWGEAGA
2454	2459	NS4B	0.0000	0.0000	6	0.3333	0.1667	0.1667	0.1667	AWGWGE
2703	2708	NS5	0.0000	0.0000	6	0.3333	0.1667	0.1667	0.1667	YTSTMM
2704	2709	NS5	0.0000	0.0000	6	0.3333	0.1667	0.1667	0.1667	TSTMME
2705	2710	NS5	0.0000	0.0000	6	0.3333	0.1667	0.1667	0.1667	STMMET
3403	3412	NS5	0.0000	0.0000	10	0.3000	0.0000	0.2000	0.0000	STQVRYLGEE
3404	3413	NS5	0.0000	0.0000	10	0.3000	0.0000	0.2000	0.0000	TQVRYLGEEG
3405	3414	NS5	0.0000	0.0000	10	0.4000	0.0000	0.2000	0.0000	QVRYLGEEGS
3408	3413	NS5	0.0000	0.0000	6	0.3333	0.0000	0.1667	0.0000	YLGEEG

aa: amino acid; DENV: dengue virus; JEV: Japanese encephalitis virus; NS: non-structural; pr: precursor; WNV: West Nile virus; YFV: yellow fever virus.

^a^ *K*-mer is the protein fragment’s length in amino acids.

Note: None of the peptides had any homology with chikungunya virus.

**Table 3:** The number of Zika virus protein fragments selected as lead candidates for developing a serological test

Protein	No. of protein fragments											
	6-mer	10-mer	20-mer	30-mer	40-mer	50-mer	60-mer	70-mer	80-mer	90-mer	100-mer	Total
Capsid C	1	0	0	0	8	1	0	6	0	0	0	16
prM	3	1	0	0	0	0	0	0	0	0	0	4
E	0	6	0	5	7	0	0	0	0	0	0	18
NS1	3	1	0	0	0	0	0	0	0	0	0	4
NS2A	4	10	7	0	3	4	0	0	0	0	0	28
NS2B	1	7	5	0	0	0	0	0	0	0	0	13
NS3	0	0	0	0	0	0	0	0	0	0	0	0
NS4A	0	0	5	0	5	14	24	44	38	32	28	190
NS4B	5	6	3	0	0	0	0	0	0	0	0	14
NS5	4	3	0	0	0	0	0	0	0	0	0	7
**Total**	**21**	**34**	**20**	**5**	**23**	**19**	**24**	**50**	**38**	**32**	**28**	**294**

E: envelope; NS; non-structural; prM; precursor membrane.

Note: mer refers to the amino acids length of the protein fragment. Candidate proteins were selected based on identity between Zika virus and other viruses, within-Zika virus polymorphism, and protein structure.

**Fig. 4:**
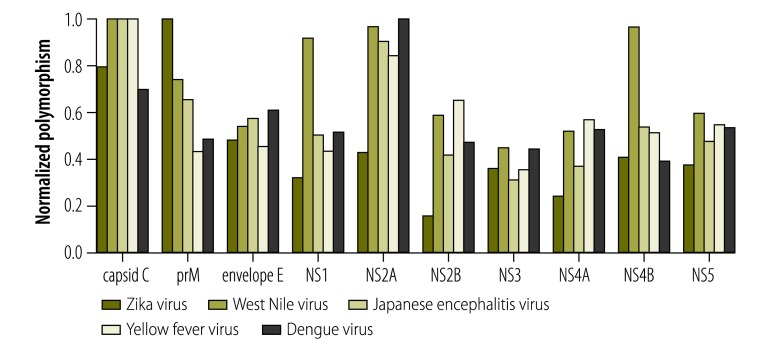
Normalized within-species polymorphism for each gene of each virus

As Zika virus infection is associated with birth defects that are not seen in other flavivirus infections, we compared identity and polymorphism of proteins between flaviviruses. Overall, the level of identity between Zika virus and other flaviviruses is similar to the level of identity seen when comparing other flaviviruses with each other (available from the corresponding author). In contrast, one region (amino acid positions 430–500 in the proteome) in the envelope protein shows both low identity between Zika virus and other flaviviruses and low polymorphism within Zika virus (Fig. 2) and the relative polymorphism of NS2A and NS2B is on average 53.6% and 69.5% lower in Zika virus than in other flaviviruses, respectively (Fig. 4).

E: envelope; NS; non-structural; prM; precursor membrane.

Notes: The proportion of polymorphic sites of each gene is normalized by the highest proportion of polymorphic sites for each virus.

**Fig. 5:**
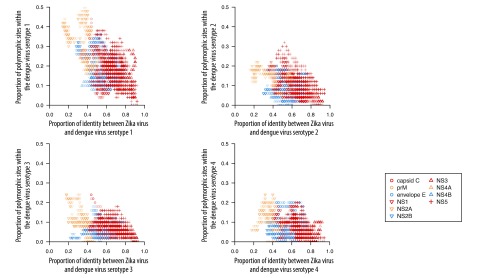
Dengue virus polymorphism versus identity with Zika virus

Protein identity between dengue and Zika viruses is negatively associated with polymorphism within the dengue virus proteins (*P*-values < 0.01 for all dengue serotypes; Fig. 5). This result can be explained by so-called negative selection, i.e. protein regions under stronger selective constraints tend to be more conserved and have higher identity between species and lower polymorphism within species.^26^ We did not observe a similar association for within-Zika virus polymorphism, which might be due to fewer strains analysed and/or smaller effective size of the global Zika virus population from which sequences were sampled, resulting in lower selection efficiency.

E: envelope; NS; non-structural; prM; precursor membrane.

Notes: 50-mers across the Dengue virus proteome were analysed, using a sliding window approach. Dengue virus polymorphism is negatively associated with the identity between dengue virus and Zika virus.

## Discussion

Here we identified regions within the Zika virus proteome that have low identity with other viruses and low within-species polymorphism. These regions may be used to develop new serological diagnostic tests to detect Zika virus infection. However, for some of the identified regions, their antigenic properties are unknown and, therefore, these regions would first need to be evaluated for such properties. The regions identified as antigenic could then be used for developing a peptide microarray, where a collection of identified peptides are displayed on a surface. Antibodies generated during a previous Zika virus infection will then be able to bind to these displayed peptides. The read-out of the microarray is the fluorescent signal generated by fluorescence-coupled secondary antibodies that have bound to the serum antibody–peptide complexes. An advantage of assessing multiple peptides simultaneously in one test is that individual peptides do not need to generate a strong signal, since the intensities of signals of all different antibody–peptide complexes can be incorporated into a composite signal. Through statistical modelling the signal generated can be used to distinguish Zika virus infection and other infections.^16^ Microarrays also have a greater potential to identify prior virus infections than neutralization-based assays, because microarrays can detect a broader range of antibodies than only antibodies that neutralize the virus and protect against infections. Peptide microarrays have been used to differentiate between serological responses to closely related bacterial pathogens^16^ and to detect previous viral infections.^27^

The computational selection strategy used here represents a targeted approach, which reduces the number of potential candidate peptides. These peptides could be used for creating a peptide–antibody signature for a given viral infection. Once the signature is identified, a diagnostic test employing only the most important peptides contributing to that signature can be designed and produced. While our computational analysis of *k*-mers focused on linear epitopes, specific and sensitive linear epitopes together may be sufficient to distinguish different arboviruses. Moreover, depending on how a serological diagnostic test is produced, some of the longer *k*-mers might fold with sufficient similarity to their native folding to present conformational epitopes.

Our analysis showed that NS1 protein polymorphism is low. Therefore, using peptides from the NS1 protein for diagnostic test might result in a high-sensitivity test for detecting antibodies against Zika virus from different geographical locations. On the contrary, the identity of NS1 protein across flaviviruses is not particularly low compared to other proteins (third highest among 10 proteins), suggesting that NS1 is not the top candidate protein for low cross-reactivity. Recently, Euroimmun AG (Lübeck, Germany) developed a Zika virus ELISA for immunoglobulins (Ig)M and IgG, based on the NS1 protein. Preliminary results show that the test is Zika virus specific.^28^^,^^29^ However, the small sample size, the fact that the samples were not from regions with endemic dengue and the lack of samples from patients with different stages of infection weaken the conclusion.^28^^,^^29^ Moreover, because each diagnostic test has its advantages and disadvantages, having multiple approaches available is helpful for providing an accurate diagnosis. A sensitive and specific diagnostic test detecting several arbovirus infections simultaneously would be valuable,^1^ so that only one assay is required to diagnose active and previous flavivirus infection(s). While we designed the sequence analysis for specificity and sensitivity of detection of Zika virus infection, the same type of analysis could be used for identifying specific and sensitive markers for each arbovirus. By including specific and sensitive markers from all arboviruses in the same peptide microarray, the microarray has the potential to detect several arbovirus infections simultaneously.

To further dissect the molecular mechanism leading to the Zika virus sequelae not seen with other flaviviruses, the protein fragments presented in the candidate list may be useful. The low polymorphisms in NS2A and NS2B proteins might be good candidates to start investigating the possible molecular link between Zika virus and microcephaly and Guillain–Barré syndrome.

Peptide-sequence identity is unlikely to fully predict cross-reactivity due to other factors, such as glycosylation. Nonetheless, this analysis based on publicly available sequences provides a step towards the development of a serological test that can distinguish previous Zika virus and co-circulating arbovirus infections.^1^
